# Epidemiological and clinical characterization of community, healthcare-associated and nosocomial colonization and infection due to carbapenemase-producing *Klebsiella pneumoniae* and *Escherichia coli* in Spain

**DOI:** 10.1007/s15010-024-02267-0

**Published:** 2024-05-04

**Authors:** Elena Salamanca-Rivera, Zaira R. Palacios-Baena, Javier E. Cañada, Zaira Moure, María Pérez-Vázquez, Jorge Calvo-Montes, Luis Martínez-Martínez, Rafael Cantón, Guillermo Ruiz Carrascoso, Cristina Pitart, Ferran Navarro, Germán Bou, Xavier Mulet, Juan José González-López, Fran Sivianes, Mercedes Delgado-Valverde, Álvaro Pascual, Jesús Oteo-Iglesias, Jesús Rodríguez-Baño, The GEMARA/GEIRAS-SEIMC/REIPI CARB–ES–19 Group, Mariela Martínez Ramírez, M. Pilar Ortega Lafont, Emilia Cercenado, Cristobal del Rosario, Jose Luis Perez Arellano, María Lecuona, Luis López-Urrutia Lorente, José Leiva, José Luis del Pozo, Salvador Giner, Juan Frasquet, Lidia Garcia Agudo, Soledad Illescas, Pedro de la Iglesia, Rosario Sánchez Benito, Eugenio Garduño, Ma Isabel Fernández Natal, Marta Arias, Mar Olga Pérez Moreno, Ana Isabel López-Calleja, José Manuel Azcona, Alba Belles, Mercè García González, Miriam Valverde Troya, Begoña Palop, Fernando García Garrote, Jose Luis Barrios Andrés, Leyre López Soria, Adelina Gimeno, Ester Clapés Sanchez, Jennifer Villa, Nuria Iglesias Nuñez, Rafael Sánchez Arroyo, Susana Hernando, Eva Riquelme Bravo, Caridad Sainz de Baranda, Oscar Esparcia Rodríguez, Jorge Gaitán, María Huertas, M. José Rodríguez Escudero, Carmen Aldea, Nerea Sanchez, Antonio Casabella Pernas, Ma Dolores Quesada, Carmina Martí Sala, Laura Mora, Encarnación Clavijo, Natalia Chueca, Federico Juan Manuel GarcíaSánchez, Fátima Galán Sánchez, Carmen Liébana, Carolina Roldán, Ma Isabel Cabeza, Ma Teresa Cabezas Fernández, Lucía Martínez Lamas, Sonia Rey Cao, Ma Isabel Paz Vidal, Raquel Elisa Rodríguez Tarazona y Noelia Arenal Andrés, Amparo Coira Nieto, Ma Luisa Pérez del Molino Bernal, María Gomáriz Díaz, Matxalen Vidal-García, Jose Luis de Tuesta Díaz, Moises García Bravo, Almudena Tinajas, Fe Tubau Quintano, Borja Suberviola Cañas y Maria Eliecer Cano García, Irene Gracia-Ahufinger, Mónica González Bardanca, Belén Viñado, Xavier Nuvials, Ignasi Roca, Patricia Ruiz-Garbajosa, Desireé Gijon, Vicente Pintado, Alba Rivera, David Gutiérrez Campos, Aurora Alemán, Ignacio Ayestarán, Andrés Canut Blasco y Jorge Arribas García

**Affiliations:** 1 Unidad de Gestión Clínica de Enfermedades Infecciosas y Microbiología, Hospital Universitario Virgen Macarena, Instituto de Biomedicina de Sevilla (IBiS), CSIC, Universidad de Sevilla, Avda Dr. Fedriani, 3, 41009 Seville, Spain; 2 CIBERINFEC, Instituto de Salud Carlos III, Madrid, Spain; 3 Laboratorio de Referencia a Antibióticos E Infecciones Relacionadas Con La Asistencia Sanitaria, Centro Nacional de Microbiología, Instituto de Salud Carlos III, Madrid, Spain; 4 Servicio de Microbiología, Hospital Universitario Marqués de Valdecilla, IDIVAL, Santander, Spain; 5 Microbiology Unit, Department of Agricultural Chemistry, Soil Science and Microbiology, Reina Sofia University Hospital, University of Córdoba, Maimonides Biomedical Research Institute of Cordoba (IMIBIC), Córdoba, Spain; 6 Servicio de Microbiología, Hospital Universitario Ramón y Cajal, Instituto Ramón y Cajal de Investigación Sanitaria (IRYCIS), Madrid, Spain; 7 Servicio de Microbiología Clínica, Hospital Universitario La Paz (IdiPAz), Madrid, Spain; 8 Servicio de Microbiología, Hospital Clínic de Barcelona, ISGlobal Barcelona Institute for Global Health, Barcelona, Spain; 9 Servicio de Microbiología, Hospital de La Santa Creu I Sant Pau. Departament de Genètica I de Microbiologia Universitat Autónoma de Barcelona (UAB), Sant Pau Biomedical Research Institute (IIB Sant Pau), Barcelona, Spain; 10 Servicio Microbiología, Hospital Universitario A Coruña, Instituto Investigación Biomédica A Coruña (INIBIC), A Coruña, Spain; 11 Servicio de Microbiología, Hospital Universitario Son Espases, Instituto de Investigación Sanitaria Illes Balears (IdISBa), Palma, Spain; 12 Servei de Microbiologia, Departament de Genetica I Microbiologia, Hospital Universitari Vall d’Hebron, Universitat Autònoma de Barcelona, Barcelona, Spain

**Keywords:** Carbapenem resistant *Enterobacterales*, Risk factors, Clinical features, OXA-48, KPC-3

## Abstract

**Background:**

Community-acquired (CA) and healthcare-associated (HCA) infections caused by carbapenemase-producing Enterobacterales (CPE) are not well characterized. The objective was to provide detailed information about the clinical and molecular epidemiological features of nosocomial, HCA and CA infections caused by carbapenemase-producing *Klebsiella pneumoniae* (CP-Kp) and *Escherichia coli* (CP-Ec).

**Methods:**

A prospective cohort study was performed in 59 Spanish hospitals from February to March 2019, including the first 10 consecutive patients from whom CP-Kp or CP-Ec were isolated. Patients were stratified according to acquisition type. A multivariate analysis was performed to identify the impact of acquisition type in 30-day mortality.

**Results:**

Overall, 386 patients were included (363 [94%] with CP-Kp and 23 [6%] CP-Ec); in 296 patients (76.3%), the CPE was causing an infection. Acquisition was CA in 31 (8.0%) patients, HCA in 183 (47.4%) and nosocomial in 172 (48.3%). Among patients with a HCA acquisition, 100 (54.6%) had been previously admitted to hospital and 71 (38.8%) were nursing home residents. Urinary tract infections accounted for 19/23 (82.6%), 89/130 (68.5%) and 42/143 (29.4%) of CA, HCA and nosocomial infections, respectively. Overall, 68 infections (23%) were bacteremia (8.7%, 17.7% and 30.1% of CA, HCA and nosocomial, respectively). Mortality in infections was 28% (13%, 14.6% and 42.7% of CA, HCA and nosocomial, respectively). Nosocomial bloodstream infections were associated with increased odds for mortality (adjusted OR, 4.00; 95%CI 1.21–13.19).

**Conclusions:**

HCA and CA infections caused by CPE are frequent and clinically significant. This information may be useful for a better understanding of the epidemiology of CPE.

## Introduction

Over the past decades, the spread of carbapenemase-producing Enterobacterales (CPE) has become a worldwide public health threat [1]. The spread of carbapenemase-encoding genes is associated both to horizontal transmission of mobile genetic elements carrying these genes between different bacteria and to transmission of successful clones [2]. Although CPE were initially identified as nosocomial pathogens, it was soon evident that nursing homes might became reservoirs for these bacteria [3], and community-acquired infections were reported [4]. However, comprehensive studies providing clinical and molecular epidemiological information of non-nosocomial infections caused by CPE are scarce [5], despite being important for infection control and clinical management purposes.

Using data from a nationwide surveillance study in Spain, we aimed at providing detailed information about the clinical and molecular epidemiological features of nosocomial, healthcare-associated and community-acquired infections caused by carbapenemase-producing *Klebsiella pneumoniae* (CP-Kp) and *Escherichia coli* (CP-Ec) isolates.

## Methods

### Study design, sites and selection of participants

This prospective cohort study is part of a multicenter and prospective cohort study (CARB-ES-19) developed in 71 Spanish hospitals between 1st February and 31st March 2019, in which the first 10 consecutive patients from whom carbapenemase-producing CP-Kp or CP-Ec were isolated in clinical samples at each of the participating hospitals were included. Patients from whom CP-Kp or CP-Ec were isolated only from surveillance samples (e.g., rectal or skin swabs) were excluded. Patients from whom *K. pneumoniae* or *E. coli* with meropenem MIC > 0.12 mg/L were eligible; only those with isolates producing carbapenemases as detailed below were finally included. The molecular characterization, resistance mechanisms, virulence genes, plasmids and antimicrobial susceptibility of the 403 isolates included were previously published [6]. In summary, 377 (93.5%) were *K. pneumoniae*; the most frequent carbapenemase genes were *bla*_OXA-48_ (263, 69.8%), *bla*_KPC-3_ (62, 16.4%) and *bla*_VIM-1_ (28, 7.4%); the most frequent sequence types (ST) among *K. pneumoniae* were ST307 (82, 21.7%), ST11 (68, 18%), ST258/512 (52, 13.8%) and ST15 (48, 12.7%).

Fifty-nine of the 71 hospitals participated in the collection of clinical data; patients from those sites are included in this analysis.

### Variables and definition

Data collected included demographics, severity of chronic conditions using Charlson index [7] and McCabe classification, specific chronic underlying diseases, type of acquisition, previous exposure to invasive procedures, travels abroad, use of antibiotic during last month, site of infection, development of severe sepsis or septic shock [8], antimicrobial therapy received (including empirical and targeted treatment) and 30-day all-cause mortality.

McCabe classification includes rapidly fatal underlying diseases if death is expected during the next year, ultimately fatal if death is expected in the next 5 years, and non-fatal otherwise. Acquisition was classified as nosocomial if the infection signs started after 48 h of admission or in less than 48 h after hospital discharge; not nosocomial cases were considered healthcare-associated if the patient had relevant healthcare contact during the last year, including previous hospital admission, nursing home residency, > 3 visits in specialized outpatient clinic, receiving intravenous ambulatory treatment, had undergone surgery or dialysis. In other case, acquisition was considered as community. Patients with clinical signs or symptoms of infection attributable to the CPE isolated in opinion of the local investigator were considered as infected; otherwise, they were considered as colonized, including patients with asymptomatic bacteriuria. The site of infection was decided by the local investigators considering the sample where the microorganism was isolated, together with focal signs and symptoms, biological markers and image tests. Antibiotic treatment was considered empirical when administered before susceptibility data were available, and active when including at least one drug with in vitro activity according to EUCAST criteria, used at recommended dosing for the corresponding MIC.

### Microbiological studies

Local laboratories used standard methods to detect candidate isolates; confirmation of carbapenamase production by PCR was performed at ten centers; then, whole genome sequencing was performed at the Antibiotic Reference Laboratory, Centro Nacional de Microbiología, Madrid, Spain. All methodological details were reported previously. [6]

### Statistical analysis

Differences between groups were compared using Chi-Square test or Fisher’s exact test for categorical variables as appropriate and the Mann–Whitney U test used for continuous variables. Logistic regression analysis was performed to study relation of the patients’ characteristics with 30-day all-cause mortality. Variables with a p value < 0.1 in the bivariate analysis and those considered of clinical importance were included in the model and selected using a stepwise backward method; the variable “acquisition type” was maintained in all the models. Interactions and collinearity between variables were considered. SPSS 25.0 was used for the analyses (IBM Corp, Armonk, NY, USA).

## Results

Overall, CP-Kp or CP-Ec were isolated from clinical samples in 403 patients; isolates from 17 patients were admitted to hospitals not participating in the clinical study, and therefore we finally included 386 patients in this analysis, of which 363 (94%) had CP-Kp and 23 (6%) CP-Ec. Overall, 296 patients (76.3%) were causing an infection (280 CP-Kp and 16 CP-Ec), and 90 (23.7%) after medical chart review were considered as colonizers (83 CP-Kp and 7 CP-Ec).

The comparison of isolates causing colonization and infection is shown in Table 1; they were similar in bacterial species, sequence types (ST) distribution, carbapenemase genes and antimicrobial resistance rates, with few exceptions: colonizing isolates somehow more frequently belonged to *K. pneumoniae* ST147 and ST392, and were more frequently resistant to meropenem/vaborbactam, plazomicin and ciprofloxacin. The most frequent type of sample where the first isolate per patient was obtained was urine; however, isolation from urine sample was more frequent in colonized patients (it was considered as asymptomatic bacteriuria in all of them) than in patients with infections, and the opposite occurred with blood and exudates.

**Table 1:** Characteristics of carbapenemase-producing *K. pneumoniae* or *E. coli* causing colonization or infection. Data are number of isolates (percentage)

	All cases (n = 386)	Colonization (n = 90)	Infection (n = 296)	P value
Microorganism				
* K. pneumoniae*	363 (94.0)	83 (92.2)	280 (94.6)	0.40
* E. coli*	23 (6.0)	7 (7.8)	16 (5.4)	
Sequence type (*K. pneumoniae*)				
ST11	67 (17.4)	16 (17.8)	51 (17.2)	0.82
ST15	47 (12.2)	15 (16.7)	32 (10.8)	0.08
ST147	36 (9.3)	14 (15.6)	22 (7.4)	0.01
ST307	77 (19.9)	16 (17.8)	61 (20.6)	0.62
ST392	15 (3.9)	7 (7.8)	8 (2.7)	0.02
ST258/512	49 (23.3)	8 (9.6)	41 (14.6)	0.24
Carbapenemase type^a^				
OXA-48-like	276 (71.5)	67 (74.4)	209 (70.6)	0.48
KPC	71 (18.4)	17 (18.9)	54 (18.2)	0.89
Metallo-β-lactamases	43 (11.1)	9 (10.0)	34 (11.4)	0.69
Antimicrobial resistance				
Ceftazidime	319 (82.6)	74 (82.2)	245 (82.8)	0.43
Meropenem	104 (26.9)	27 (30.0)	77 (26.0)	0.45
Aztreonam	309 (80.1)	75 (83.3)	234 (79.1)	0.37
Ceftazidime/avibactam	98 (25.4)	20 (22.2)	78 (26.4)	0.43
Meropenem/vaborbactam	39 (10.1)	15 (16.7)	24 (8.1)	0.01
Imipenem/relebactam	84 (21.8)	22 (24.4)	62 (20.9)	0.48
Cefiderocol	11 (2–8)	2 (2.2)	9 (3.0)	0.68
Gentamicin	179 (46.4)	46 (41.1)	133 (44.9)	0.30
Tobramycin	272 (70.5)	66 (73.3)	206 (69.6)	0.46
Amikacin	72 (18.7)	21 (23.3)	51 (17.2)	0.19
Plazomicin	23 (6.0)	14 (15.6)	9 (3.0)	< 0.001
Tigecycline	24 (6.4)	5 (5.6)	19 (6.4)	0.90
Ciprofloxacin	358 (92.7)	88 (97.8)	270 (91.2)	0.03
Fosfomycin	209 (54.1)	52 (57.8)	157 (53.0)	0.43
Colistin	37 (9.6)	5 (5.6)	32 (10.8)	0.13
Trimethoprim-sulfamethoxazole	265 (68.7)	63 (70.0)	202 (68.2)	0.75
Sample (first isolate)				< 0.001
Urine	206 (53.4)	74 (82.2)	132 (44.6)	
Respiratory	45 (11.7)	7 (7.8)	38 (12.8)	
Vascular catheter	9 (2.3)	3 (3.3)	6 (2.0)	
Exudate	43 (11.1)	2 (2.2)	41 (13.9)	
Blood	50 (13.0)	0	50 (16.9)	
Others	33 (8.5)	4 (4.4)	29 (9.8)	

^a^Seven isolates produced two carbapenemases

The features of the patients with infection or colonization are shown in Table 2. The median age of patients was 74 years, and 181 (46.9%) were women, and had a median Charlson index of 2. The most frequent comorbidities were diabetes mellitus, chronic heart insufficiency and solid cancer; hemiplegia and immunosuppression were more frequent among colonized than infected patients. Interestingly, acquisition was community-onset but healthcare-associated in 183 patients (47.4%) and strict community-associated in 31 (8.0%). Overall, nosocomial acquisition was more frequent in infected than colonized patients, while in colonized patients, healthcare-associated acquisition was more frequent. Among patients with a healthcare-associated acquisition, previous hospital admission and being nursing home resident were frequent; the latter was more frequent in colonized than infected patients. In patients with nosocomial acquisition, most were admitted to medical wards, and had a long previous hospitalization before the isolate was obtained (median, of 21 days). Previous invasive procedures and antibiotic use were frequent in both colonized and infected patients; the most frequent previous antibiotics were carbapenems and fluoroquinolones; piperacillin-tazobactam was more frequent among patients with infection than among colonized. Finally, mortality was more frequent among patients with infection.

**Table 2:** Characteristics and epidemiological features of patients with colonization or infection due to carbapenemase-producing *K. pneumoniae* or *E. coli*. Data are number (percentage) of patients except where specified

	All patients (n = 386)	Colonization (n = 90)	Infection (n = 296)	P value
Median age in years (IQR)	74 (64–85)	77 (62–86)	74 (64–84)	0.39
Female sex	181 (46.9)	46 (51.1)	135 (45.6)	0.36
Underlying conditions				
Diabetes mellitus	128 (33.2)	28 (31.1)	100 (33.8)	0.63
Chronic pulmonary disease	66 (17.1)	15 (16.7)	51 (17.2)	0.90
Chronic heart insufficiency	96 (24.9)	27 (30.0)	69 (23.3)	0.19
Chronic renal insufficiency	80 (20.7)	21 (23.2)	59 (19.9)	0.48
Chronic liver disease	27 (7.0)	5 (5.6)	22 (7.4)	0.54
Dementia	58 (15.0)	17 (18.9)	41 (13.9)	0.24
Hemiplegia	23 (6.0)	11 (12.2)	12 (4.1)	0.004
Solid cancer	97 (25.1)	18 (20.0)	79 (26.7)	0.20
Hematologic cancer	17 (4.4)	1 (1.1)	16 (5.4)	0.13
Immunosuppression	49 (12.7)	18 (20.0)	31 (10.5)	0.01
Charlson index, median (IQR)	2 (1–4)	2 (1–4)	2 (1–4)	0.64
Acquisition				0.02
Community	31 (8.0)	8 (8.9)	23 (7.8)	
Healthcare-associated	183 (47.4)	53 (58.9)	130 (48.3)	
Nosocomial	172 (48.3)	29 (32.2)	143 (48.3)	
Type of healthcare contact (healthcare-associated cases)				
Hemodialysis	6 (3.3)	2 (3.8)	4 (3.1)	> 0.99
Ambulatory IV therapy	14 (7.7)	3 (5.7)	11 (8.5)	0.76
> 3 visits to specialized clinic	46 (25.1)	11 (20.8)	35 (26.9)	0.75
Previous admission	100 (54.6)	28 (52.8)	72 (55.4)	0.75
Nursing home resident	71 (38.8)	29 (54.7)	42 (32.3)	0.005
Ward of admission (nosocomial cases)				0.17
Medical ward	85 (49.1)	19 (63.3)	66 (46.2)	
Surgical ward	43 (24.9)	4 (13.3)	39 (27.3)	
Intensive care	45 (26.0)	7 (23.3)	38 (26.6)	
Median days of previous hospitalization (IQR), nosocomial cases	21 (11–39)	24 (10–31)	21 (11–40)	0.91
Travel abroad last year^a^	8 (2.1)	2 (2.2)	6 (2.0)	> 0.99
Invasive procedures^b^				
Bladder catheter	159 (41.2)	36 (40.0)	123 (41.6)	0.79
Vascular catheter	137 (35.5)	84 (26.7)	113 (38.2)	0.04
Surgery (previous month)	82 (21.2)	19 (21.1)	63 (21.3)	0.97
Mechanical ventilation	48 (12.4)	12 (13.3)	36 (12.2)	0.76
Endoscopic procedure	28 (7.3)	6 (6.7)	22 (7.4)	0.80
Antibiotics received (last month)				
Any	299 (77.5)	67 (74.4)	232 (78.4)	0.43
Cephalosporins	94 (24.4)	25 (27.8)	69 (23.3)	0.38
Piperacillin/tazobactam	50 (13.0)	6 (6.7)	44 (14.9)	0.04
Carbapenems	88 (22.8)	15 (16.7)	73 (24.7)	0.11
Fluoroquinolones	88 (22.8)	17 (18.9)	71 (24.0)	0.31
Mortality	91 (23.6)	8 (8.9)	83 (28.0)	< 0.001

^a^Travel to countries in: South America, three patients; Africa, two patients; Asia, two patients; Easter Europe, one patient

^b^In the previous week except where specified

IV: intravenous

Regarding the 296 patients with infection, the data are shown in Table 3. *K. pneumoniae* was less frequent in community-acquired infections. The most frequent was urinary tract (50.7%), followed by respiratory tract (14.2%). Overall, 68 infections (23%) were bacteremic. When classified according to acquisition, infection due to *K. pneumoniae* was less frequent in community-acquired cases; urinary tract infections (UTI) were more frequent in community and healthcare-associated infections than in nosocomial ones, while the opposite occurred with respiratory tract and intraabdominal infections. Bacteremia, development of severe sepsis or shock and receipt of active empirical therapy were more frequent in nosocomial infections and less in community-acquired ones. Because of its frequency, UTI were stratified; overall, community-acquired UTI were more frequently afebrile and not associated with urinary devices, and nosocomial episodes were more frequently febrile and associated with devices. Finally, mortality was higher in nosocomial episodes than in community or healthcare associated.

**Table 3:** Features of patients with infections caused by carbapenemase-producing *K. pneumoniae or E. coli*, according to type of acquisition

	All infections (n = 296)	Community (n = 23)	Healthcare-associated (n = 130)	Nosocomial (n = 143)	P value
Microorganism: *K. pneumoniae*	280 (94.6)	19 (81.6)	127 (97.7)	134 (93.7)	0.010
Carbapenemase type^a^					
OXA-48-like	209 (70.6)	18 (78.3)	95 (73.1)	96 (67.1)	0.39
KPC	54 (18.2)	2 (8.7)	25 (19.2)	27 (18.9)	0.46
Metallo-β-lactamases	34 (11.5)	2 (8.7)	10 (7.7)	22 (15.4)	0.12
Site of infection					< 0.001
Urinary	150 (50.7)	19 (82.6)	89 (68.5)	42 (29.4)	
Respiratory	42 (14.2)	1 (4.3)	8 (6.2)	33 (23.1)	
Soft tissues	38 (12.8)	2 (8.7)	13 (10.0)	23 (16.1)	
Intraabdominal	33 (11.1)	0	8 (6.1)	25 (17.5)	
Catheter	6 (2.0)	0	2 (1.5)	4 (2.8)	
Bacteremia, unknown source	21 (7.1)	1 (4.3)	7 (5.4)	13 (9.1)	
Others	4 (1.4)	0	2 (1.5)	2 (1.4)	
Bloodstream infection, all sources	68 (23.0)	2 (8.7)	23 (17.7)	43 (30.1)	0.012
Surgical site infection	23 (7.8	0	7 (5.4)	16 (11.2)	0.071
Types of urinary tract infection					< 0.001
Afebrile, no device-associated	54 (36.0)	16 (84.2)	33 (37.1)	5 (11.9)	
Afebrile, device-associated	21 (14.0)	0	16 (18.0)	5 (11.9)	
Febrile, no device-associated	33 (22.0)	3 (15.8)	18 (20.2)	12 (28.6)	
Febrile, device-associated	42 (28.0)	0	22 (24.7)	20 (47.6)	
Severe sepsis or shock	54 (18.2)	2 (8.7)	14 (10.8)	38 (26.6)	0.002
Active empirical therapy	55 (18.5)	2 (8.7)	17 (13.0)	36 (25.1)	0.02
Mortality	83 (28.0)	3 (13.0)	19 (14.6)	61 (42.7)	< 0.001

^a^Three isolates produced two carbapenemases

The impact of acquisition in mortality was analyzed in the 68 patients with bacteremia, of which 2 (2.9%) were community-acquired, 23 (33.8%) were healthcare-associated and 43 (63.2%) had a nosocomial acquisition. Mortality rates according to acquisition were 1/2 (50%), 17/23 (30.4%) and 26/43 (60.5%) in community, healthcare-associated and nosocomial episodes, respectively. Because of the low number of community-acquired bloodstream infections, the variable “acquisition type” was dichotomized into nosocomial and non-nosocomial cases (the latter including healthcare-associated and community-acquired cases). Because only two bacteremic cases were caused by *E. coli*, we did not consider the variable “microorganism” in this analysis. In the univariate analysis, isolates producing MBL, patients with fatal underlying disease, mechanical ventilation and high-risk sources (i.e., other than urinary tract, intraabdominal and soft tissue infections) showed a p value for their association with death < 0.1 (Table 4) and were included in the logistic regression model; presentation with severe sepsis or shock was not considered as being in the pathogenic pathway to mortality. In the final model, nosocomial bloodstream infections were associated with increased odds for mortality (adjusted OR 4.00; 95% CI 1.21–13.19; p = 0.0022) (Table 4). The number of patients treated with different antibiotic regimens was too low to perform specific analyses.

**Table 4:** Bivariate and multivariate analysis of the impact of acquisition type in mortality among patients with bacteremia due to carbapenemase-producing *K. pneumoniae* or *E. coli*

Characteristic		Dead/exposed (%)	OR (95% CI)	P value	Adjusted OR (95% CI)	P value
OXA-48 carbapenemase	Yes	21/47 (44.7)	0.72 (0.45–1.14)	0.18	–	–
	No	13/21 (61.9)	Ref			
KPC carbapenemase	Yes	9/16 (56.3)	1.17 (0.69–1.96)	0.56	–	–
	No	25/52 (40.0)	Ref			
MBL carbapenemase	Yes	5/6 (83.3)	1.78 (1.14–2.85)	0.08	–	–
	No	29/62 (46.8)	Ref			
High-risk clone	Yes	26/51 (51.0)	1.01 (0.61–1.92)	0.77	–	–
	No	8/17 (47.1)	Ref			
Age in years	< 65	14/24 (58.3)	0.78 (0.49–1.25)	0.31	–	–
	≥ 65	20/44 (45.5)	Ref			
Sex	Female	17/38 (44.7)	0.78 (0.49–1.26)	0.32	–	–
	Male	17/30 (56.7)	Ref			
Fatal underlying disease	Yes	25/69 (64.1)	2.08 (1.14–3.84)	0.007	6.20 (1.85–20.70)	0.003
	No	9/29 (31.0)	Ref			
Mechanical ventilation	Yes	12/15 (80.9)	1.96 (1.28–2.94)	0.008	–	–
	No	22/53 (41.5)	Ref			
High-risk source	Yes	22/35 (62.9)	1.57 (1.03–4.16)	0.02	3.81 (1.20–12.10)	0.023
	No	12/33 (36.4)	Ref			
Active empirical therapy	Yes	8/19 (42.1)	0.79 (0.44–1.44)	0.41	–	-
	No	26/49 (53.1)	Ref			
Nosocomial acquisition	Yes	26/43 (60.5)	1.92 (1.02–3.57)	0.02	4.00 (1.21–13.19)	0.022
	No	8/25 (32.0)	Ref			

## Discussion

In this study, we characterized the microbiological and clinical features of patients from whom CPE were isolated from clinical samples in a multicenter study in Spain. Overall, in around 1/4 of cases, the CPE isolate was not considered to be causing an infection; healthcare-associated acquisition was frequent, mostly in relation with previous hospital admission or nursing home residency. UTI was the most frequent type of infection but predominated mostly in community-acquired and healthcare-associated cases, while respiratory tract infections and bacteremia were more frequent in nosocomial cases. Nosocomial acquisition was associated with borderline increased risk of mortality after controlling for confounders.

A literature review published in 2017 found 15 studies providing some data about community-associated carbapenem-resistant Enterobacterales cases (not just CPE) [5]. While no community-associated CRE cases were found in 5 studies, the proportion of community-associated or community-onset cases was 0.04% to 29.5% in the other 10 studies. However, because the definitions used for community-associated cases were heterogeneous, it is unclear in many of the studies whether acquisition could be strictly considered as community-acquired or healthcare-associated. In a study conducted in Madrid, Spain from 2010 to 2014 and including 780 CPE, the authors found 22.9% of cases to be community-onset, with some 13.4% not having any clear previous healthcare contact. [4]

Compared to data from a previous multicenter study performed in Spain in 2013 [9], the proportion of patients with a healthcare-associated and community acquisition of a CPE increased from 37 and 3% to 47.4% and 8%, respectively. In both studies, *bla*_OXA-48-like_ were the most frequent group of carbapenemase genes found in non-nosocomial cases. Overall, the age and comorbidities of the patients were similar in both studies, and exposure to invasive procedures was in general somehow less frequent in this study, in relation to the lower proportion of nosocomial cases. The overall mortality rates were similar (20.4% in 2013, 23.6% in this study). Among patients with infection, the frequency of UTI and respiratory tract infections were similar in both studies, but bacteremia was more frequent in this study (23 vs 10.3%). The rate of acquisition types was also similar in a multinational study performed in southern European countries, in which 7.7%, 33.6% and 58.7% of 235 patients with infection due to CRE had a community, healthcare-associated and nosocomial infection, respectively. [10]

Overall, these data suggest that the frequency of community-onset infections caused by CPE, both healthcare-associated or not, may be increasing. Despite previous healthcare contact does necessarily imply that acquisition occurred during healthcare, the type of previous healthcare contact included in the definition used in this study for healthcare-associated CPE (nursing home residents, previous hospital admission, hemodialysis, ambulatory intravenous therapy or frequent visits at specialized outpatient clinic) strongly suggest that acquisition might have occurred predominantly in that context. Of course, CPE may be more spread in the community than is apparent in studies including clinical samples because patients without any kind of healthcare contact are also at lower risk of developing infections; in fact, it is reasonable to expect CPE to behave similarly to ESBL-producing Enterobacterales, for which transmission from hospital-discharged colonized patients to their household members occurred at a rate of 1.06 and 0.65 cases per 100 person-weeks for ESBL-producing *E. coli* and *K. pneumoniae*, respectively, and that assistance for urinary and fecal excretion significantly increased the risk of transmission [11]. To our knowledge, there are scarce data from studies systematically assessing household transmission of CPE. A small study including 10 recently discharged patients colonized with CPE and 14 household contacts found a 10% probability of transmission (95% CI 4–26%). [12]

Overall, the urinary tract was the most common site of infections in all acquisition types, but their frequency was very different, ranging from 29.4% in nosocomial infections to 82.6% in community-acquired ones. Also, the type of UTI differed: community-acquired UTIs were predominantly non-febrile and not associated with urinary devices, and the opposite occurred in nosocomial UTIs; healthcare-associated UTIs had an even distribution of febrile and device-associated episodes. Similarly, considering all infections, bacteremia and mortality were more frequent in nosocomial episodes. Although when only bacteremic patients were considered, nosocomial infections were still associated with increased adjusted odds of death, it should be noted that all-cause mortality was considerable among patients with non-nosocomial infections. Active empirical therapy was very infrequent in non-nosocomial infections; this seems logical as it is very difficult to suspect CPE as a cause of community-onset infections except if previously known to be colonized by CPE [10]. Therefore, rapid microbiological test would be of extreme importance in patients presenting with severe infection.

This study has ations; the results may not be applicable to areas with a different epidemiology of carbapenemases and predominant STs; we did not study other Enterobacterales than *K. pneumoniae* and *E. coli*; and despite a careful collection of previous healthcare contact of patients with community-onset infections, there is a possibility of misclassification. Another limitation of this manuscript is that the data are from 5 years ago and we are aware of how quickly the epidemiology in reference to antimicrobial resistance evolves.

In summary, we provided detailed comprehensive characterization of patients from whom CPE was isolated in clinical samples in Spain, according to acquisition type. This information may be useful for a better understanding of the epidemiology of CPE.

## Data Availability

Data is available upon request to the corresponding author.
